# Diabetic retinopathy in Swaziland

**Published:** 2015

**Authors:** Helen Burn, Jonathon Pons

**Affiliations:** Academic Infectious Disease Trainee and Foundation Year Two Doctor: Brighton and Sussex University Hospital Trust, UK. hburn88@gmail.com; Ophthalmologist and Project Director: The Good Shepherd Eye Clinic, GSH, Siteki, Swaziland. jono@goodshepherdhosp.org

It is estimated that between 2010 and 2030 there will be a 98% increase in the number of adults in sub-Saharan Africa with diabetes.^1^ This is just one aspect of the epidemic of non-communicable diseases facing sub-Saharan Africa, driven by urbanisation, ageing, and changes to lifestyle and environment. The diabetes epidemic poses a significant challenge to health services, as non-communicable conditions should be managed by multi-disciplinary teams, with prevention as a primary aim.

Diabetes causes visual loss through early cataract formation and diabetic retinopathy (DR). These are the second and sixth leading causes of global visual impairment.^2^ DR is considered to be proliferative when abnormal blood vessels grow from the retina on to the posterior surface of the vitreous gel. Without treatment, 50% of patients with proliferative DR will be blind within 5 years.^3^

The prevalence of diabetes in the Kingdom of Swaziland is estimated to be 3.7% among the adult population^4^, and this is predicted to rise substantially in the next decade. There are currently no data on the national prevalence or severity of DR.

This article describes the results of a retrospective review of patients presenting in the 18 month period from Jan 2012 to June 2013 at the two main eye clinics in Swaziland: Good Shepherd Hospital, Siteki and St Theresa's Eye Clinic, Manzini. Together, these clinics provide ophthalmology services to approximately 75% of the population of Swaziland.

The paper clinic notes were reviewed and patients with a diagnosis of DR were included in the study. The age, sex, visual acuity, random blood sugar, classification of severity, management and co-morbidities were recorded.

## Findings

Of the 82 patients with DR, 47 were female and 35 male. The mean age was 59 with the majority of patients were aged between 50–69 years.

The best corrected visual acuity of patients with diabetic retinopathy presenting at the clinic for the first time is shown in Table 1. A total of 31 (38%) had a visual acuity of 3/60 or less in their better eye. Of these, over half had a visual acuity of counting fingers or less.

**Table 1.** Best corrected visual acuity at presentation in 81 patients with diabetic retinopathy in Swaziland

Visual acuity	Number of patients	Percentage of patients
6/6–6/18	19	23%
<6/18–6/60	23	28%
<6/60–3/60	10	12%
<3/60-NLP	29	36%
Total	81	100%

Classification of the severity of retinopathy was provided by written descriptions of retinal pathology by ophthalmologists following examination with a 90D lens. 76% of patients had proliferative diabetic retinopathy at the time of presentation.

Of the 82 patients included in the study, 41 had undergone a random blood glucose measurement at the time of presentation. 84% of these patients had random blood glucose greater than 8.0 mmol/L and 24% had a blood glucose above 16 mmol/L. A total of 56 patients (68%) had a co-existing diagnosis of hypertension.

## Discussion

It is known that duration of diabetes, poor glycaemic control, and coexisting hypertension all contribute to an increased risk of DR. The late presentation of patients with DR, and the low visual acuity of those presenting, are both strong arguments for the implementation of a DR screening programme in Swaziland. The programme would aim to reduce loss of vision caused by DR and raise awareness of the importance of strict glucose and blood pressure control in preventing complications of diabetes.

## Next steps

We intend to carry out a pilot study of retinal screening for DR in Swaziland. We plan to use retinal photographs taken in clinic, interpreted remotely by ophthalmologists, to screen the population and organise laser treatment or follow up for each individual. We acknowledge many barriers to screening, including:

- Low numbers of ophthalmic personnel in the country
- Identification and recruitment of patients
- Increased burden on existing services
- Lack of equipment.

However, it is clear that improving services for patients with diabetes and its complications is an urgent priority in order to prevent visual impairment in this population.
